# Ki-67 and Its Relation With Complete Pathological Response in Patients With Breast Cancer

**DOI:** 10.7759/cureus.16788

**Published:** 2021-07-31

**Authors:** Sana Wajid, Fauzia A Samad, Abdus S Syed, Faiza Kazi

**Affiliations:** 1 Oncology, Fauji Foundation Hospital, Rawalpindi, PAK; 2 Department of Radiation Oncology, Combined Military Hospital, Rawalpindi, PAK; 3 Pathology, Fauji Foundation Hospital, Rawalpindi, PAK

**Keywords:** ki-67, breast cancer

## Abstract

Introduction

Ki-67 is a nuclear antigen present in the synthesis phase of the cell cycle. Studies have shown that a high value of Ki-67 results in greater response to chemotherapy with higher incidence of complete pathological response, which ultimately results in improved overall survival.

Methods and materials

The objective of the study was to determine the frequency of high Ki-67 levels in breast cancer patients and to find the correlation of complete pathological response in breast cancer with Ki-67 levels. It is a descriptive case series with a correlational study design done at Fauji Foundation Hospital Rawalpindi. Eighty patients with locally advanced breast cancer who underwent neoadjuvant chemotherapy followed by surgery were recruited. Their Ki-67 levels were determined on trucut biopsy. Pathological response in the post-op sample was correlated with Ki-67 levels.

Results

The results showed 27 (33%) patients out of the 80 had high Ki-67 values. Among them 17 (63%) had complete pathological response, seven (26%) showed partial pathological response whereas three (11%) had disease progression. In contrast, out of the 53 patients having low Ki-67 values, only nine (17%) had complete pathological response, 31 (58%) showed partial pathological response and 13 (25%) had progressive disease. A Chi-square test was applied which showed significant correlation between Ki-67 and complete pathological response, with a p value of 0.00018.

Conclusion

Therefore high Ki-67 values in patients with breast cancer correlated well with attainment of complete pathological response. We can incorporate Ki-67 in the initial clinical assessment of breast cancer patients to help predict effectiveness as well as response to chemotherapy.

## Introduction

Breast cancer is the most common malignancy in women across the world. With the advent of new treatment strategies and a multidisciplinary approach, 70 to 80% of patients diagnosed with early-stage breast cancer are being cured. Of those who have locally advanced or metastatic disease, targeted therapy has played a remarkable role in improving the quality of life as well as overall survival [1,2].

Many new ongoing trials are looking into biological markers to better evaluate and categorize individuals who will obtain the maximum benefit from either chemotherapy or targeted therapy or both in early as well as advanced-stage disease.

Some of the most astounding examples in front of us are estrogen receptors, progesterone receptors and Her2/Neu receptors whose response to hormonal and targeted therapy has immensely changed the treatment approach and has improved survival rates significantly [3-6].

It is because of these results that many other biological markers are being looked into so that further understanding of the disease, as well as individualization of treatment options, can take place.

Some of the markers currently under investigation are TP53, Bcl-2 and Ki-67. Out of these biological markers, Ki-67 is of particular interest [7-9].

Ki-67 is a nuclear antigen discovered in 1980. It is expressed in active phases of the cell cycle G1, S, G2 and M phase and is absent in the quiescent phase (G0). It can be used to assess the growth fraction of a certain cell population. It can, therefore, be called a “proliferative marker of the cells” [10].

Numerous studies evaluating the percentage of Ki-67 in tumor cells and response to chemotherapy have been conducted in different malignancies such as non-Hodgkin lymphoma, bladder cancer, endometrial cancer, cervical cancers, neuroendocrine tumors and sarcomas [11-13]. These studies have shown that the greater the percentage of Ki-67, the greater is the response to chemotherapy which ultimately leads to improved overall survival.

Studies have also been conducted to evaluate its role in breast cancer. These studies have indicated that a high percentage of Ki-67 corresponds to a better response in reducing the size or completely eliminating the tumor that leads to increase in survival [14-16].

The definition of complete pathological response is resolution of invasive carcinoma in both breast tissue as well as in the lymph nodes resected in the histopathology specimen of Modified Radical Mastectomy (MRM) [5,17]. It plays a significant role in increasing disease-free survival in breast cancer patients and also has a potential role in the improvement of long-term survival as indicated by various studies [18].

Despite the numerous published papers evaluating the predictive significance of Ki-67, it is still not considered as a validated marker to be used in clinical practice as most of the studies are retrospective and small. Therefore a definite need was felt for a prospective study with sufficient number to identify any significant statistical difference. Therefore, this study was designed to evaluate the potential of Ki-67 in predicting response to chemotherapy in neoadjuvant settings. The study results can identify patients who would respond to chemotherapy early on in the treatment and thus should be subjected to rigorous chemotherapy to achieve a complete pathological response before surgery. This may translate into improved survival. Moreover, on the other hand, identification of poor responders to chemotherapy can help us decide and plan about early surgical interventions and incorporation of targeted therapies in the neoadjuvant regimens. This later decision becomes more important in a developing country like Pakistan, which has less financial resources and a high burden of breast cancer disease.

## Materials and methods

Study design

This is a descriptive case series of 80 stage III breast cancer patients conducted at Fauji Foundation Hospital, Rawalpindi, Pakistan. The duration of the study was one year, starting from January 20, 2017, till January 21, 2018. This study included female patients of histologically proven cancer of the breast aged between 18 to 65 years. All of them had stage III disease with a good performance status and normal lab results and ECHO. Pregnant patients were excluded from the study 

Method

Eighty patients having locally advanced cancer were recruited from the inpatient and outpatient department of Oncology at Fauji Foundation Hospital, Rawalpindi, from January 2017 till January 2018. Informed consent was taken from all the patients.

Patients underwent trucut biopsy of their breast lump to confirm their diagnosis of breast cancer. They also underwent staging workup to rule out distant metastasis which included blood complete picture, liver function tests, renal function tests, echocardiography and computed tomography scans of chest and upper abdomen with contrast enhancement.

Tumor block samples obtained from the trucut biopsy were evaluated for the levels Ki-67 at the histopathology department of Fauji Foundation Hospital using immunohistochemistry technique. The tumor samples were also routinely assessed for estrogen progesterone and Her2/neu receptors.

Before the start of chemotherapy, the primary breast tumor size and the axillary lymph nodes' status were clinically determined and recorded. All patients underwent six cycles of anthracycline-based chemotherapy and their tumor size was clinically evaluated after every two cycles and at the end of chemotherapy before surgery.

After the completion of chemotherapy patients were referred to the surgical department of Fauji Foundation Hospital, Rawalpindi. Patients underwent modified radical mastectomy and their samples were sent to the histopathological department of Fauji Foundation Hospital, Rawalpindi, for evaluation of any residual tumor. The pathological response of chemotherapy in histopathology report of MRM was compared with the levels of Ki-67 obtained from the trucut biopsy of the patients.

The cut-off value of Ki-67 was taken as 35% with high Ki-67 being more than 35%.

Data analysis

Data was entered and analyzed in SPSS version 16 (SPSS Inc., Chicago, IL, USA). Mean and standard deviation were calculated for quantitative variables like age, tumor size and percentage of Ki-67. Frequency and percentage were calculated for qualitative variables like gender, lymph node, stage and pathological complete response.

Chi-squared test was used to compute the association between high percentage of ki-67 and response to neoadjuvant chemotherapy. A p-value <0.05 was considered significant.

## Results

Ki-67 values were measured in the trucut biopsy samples of the patients and their response to chemotherapy was determined from the histopathology report after they underwent modified radical mastectomy.

Descriptive statistics of age (years) and tumor size (cm) were also calculated in terms of mean and standard deviation. The mean age in this study was 52.16 + 8.76 and the mean clinical tumor size (cm) was 7.71 + 3.88 (Table 1).

**Table 1:** Descriptive statistics of tumor size (cm) and age (years).

	Mean	Std. Deviation
Tumor Size (cm)	7.71	3.88
Age (years)	52.16	8.76

The percentage of low Ki-67 was higher as compared to the percentage of high Ki-67 in the study population. Fifty-three patients out of 80 patients had low Ki-67 values which made up around 66.3% of the total sample size, whereas 27 patients out of 80 patients had high Ki-67 values which made up around 33.8% of the total sample size as shown in Table 2. 

**Table 2:** Distribution of Ki-67 value in patients with locally advanced breast cancer

Ki-67	Frequency	Percentage
LOW(≤35)	53	66.3
HIGH(>35)	27	33.8
Total	80	100

Distribution of response of chemotherapy as evaluated from the histopathology report was also calculated in terms of frequency and percentage of the patients with complete pathological response, partial pathological response and progression of the disease. It was shown that 26 (32.5%) patients out of 80 had a complete pathological response whereas 38 (47.5%) patients out of 80 had a partial pathological response and 16 (20%) out of 80 had progression of the disease as shown in Table 3.

**Table 3:** Distribution of response of chemotherapy as evaluated from the histopathology report

Response	Frequency	Percentage
Complete pathological response	26	32.5
Partial Pathological response	38	47.5
Progression of disease	16	20.0
Total	80	100

The objective of this study was to determine the frequency of high values of Ki-67 in locally advanced breast cancer patients and to compare the frequency of complete pathological response with low and high values of Ki-67. The outcome of this objective was that 27 patients out of the 80 had high Ki-67 value and 17 out of them achieved a complete pathological response, with seven having a partial pathological response whereas only three had progression of the disease with high Ki-67 values.

In contrast out of the 53 patients having a low Ki-67 value only nine had complete pathological response, with 31 resulting in a partial pathological response and 13 of the 53 patients ended up having progression of the disease. Chi-square test was applied to compare the frequency of complete pathological response with high and low values of Ki- 67 which came as significant with a p-value of 0.00018 as shown in Table 4.

**Table 4:** Frequency of complete pathological response with low and high values of Ki-67

Percentage of Ki-67	Total Cases	Response			P-value
		Complete Pathological response (pCR)	Partial pathological response (pPR)	Progression (PD)	0.00018
≤35% (low)	53	9	31	13	
>35% (high)	27	17	7	3	
Total	80	26	38	16	

Effect modifiers like age were stratified and compared with the response of chemotherapy in breast cancer patients. It was seen that out of 27 patients who were less than 45 years of age, 12 had complete pathological response and partial pathological response each whereas three patients had progression of the disease. In patients who were equal to or more than 45 years of age, 14 had complete pathological response whereas 26 had partial pathological response, while 13 patients had progression of the disease. Chi square test was applied to identify any positive correlation between the age and the pathological responses and it was insignificant with a p-value of 0.1 as shown in Table 5 below.

**Table 5:** Effect modifier like age was stratified and compared with the response of chemotherapy

AGE	RESPONSE				P-value
	Complete Pathological response (pCR)	Partial Pathological Response (pPR)	Progression (PD)	Total	
<45	12	12	3	27	0.1
≥45	14	26	13	53	
Total	26	38	16	80	

Effect modifier like tumor size was stratified and compared with the responses of chemotherapy. The tumor size was divided into two categories with the size starting from 1 cm to going till 20 cm. It was seen that out of the 69 patients with the clinical tumor size of 1-10 cm, 22 had a complete pathological response while 35 had a partial pathological response, whereas 12 had progression of the disease. Out of the 11 patients who had a tumor size between 11-20 cm, four patients had a complete pathological response, while three had a partial pathological response whereas four had progression of the disease. Chi square test was applied to compare tumor size with response which came as insignificant with a p-value of 0.24 as shown in Table 6. All patients had Stage III disease so stage was not stratified.

**Table 6:** Effect modifier like tumor size was stratified and compared with the response of chemotherapy.

Clinical tumor size (cm)	Response				P- value
	Complete Pathological Response (pCR)	Partial Pathological Response (pPR)	Progression (PD)	Total	
1-10	22	35	12	69	0.24
11-20	4	3	4	11	

Stratification with respect to age was done for response of tumor with high and low values of Ki-67 which showed that the total number of patients below the age of 45 years were 27 in which high Ki-67 was seen in 10 and low Ki-67 was seen in 17 of the patients. Among the patients with high Ki-67, eight had complete pathological response which was statistically significant. The total number of patients who were 45 years or above was 53 of which 17 had a high Ki-67 value and 36 had a low Ki-67 value. Among those with high Ki-67, nine had complete pathological response which was also statistically significant. Therefore it can be inferred that Ki-67 was an independent predictor of response irrespective of age with a p value of 0.015 in patients below 45 years of age and a p value of 0.01 for patients above 45 years as shown in Table 7.

**Table 7:** Stratification with respect to age for response with High and low values of Ki-67

Age	Ki 67 %	PCR	PPR	PD	Total	p-value
<45	High	8	2	0	10	0.015
	Low	4	10	3	17	
>45	High	9	5	3	17	0.010
	Low	5	21	10	36	
Total		26	38	16	80	

Stratification with respect to size was done for response of tumor with high and low values of Ki-67 which showed that the total number of patients with a tumor size of 1-10 cm was 69, in which high Ki-67 was seen in 22 and low Ki-67 was seen in 47. The total number of patients who had a tumor size of 11-20 cm was 11, in which five had a high Ki-67 value and six patients had a low Ki-67 value. Ki-67 relationship with complete pathological response was found out to be statistically significant with a p value of 0.004 for the 1-10 cm tumor size group and a p value of 0.016 for patients who had a size of 11-20 cm as shown in Table 8. It can therefore be inferred that Ki-67 was an independent predictor of response irrespective of size.

**Table 8:** Stratification with respect to size for response with high and low values of Ki-67

SIZE	Ki67	PCR	PPR	PD	TOTAL	P VALUE
1-10	HIGH	13	6	3	22	0.004
	LOW	9	29	9	47	
11-20	HIGH	4	1	0	5	0.016
	LOW	0	2	4	6	
Total		26	38	16	80	

Stage was not stratified with response since all the patients in this study had stage III disease.

Hormone receptor status was also evaluated in relation to Ki-67 and response and the results showed that Ki-67 was a significant predictor of response in both hormone receptor positive and negative patients (Tables 9, 10).

**Table 9:** Relation of Ki-67 and Response in Hormone Receptor Positive Patients

Hormone Receptor Positive		Complete Response	Partial response	Progression	Total	P value
	High Ki-67	8	3	1	12	0.001
	Low Ki-67	3	23	4	30	
Total		11	26	5	42	

**Table 10:** Relation of Ki-67 and Response in Hormone Receptor Negative Patients.

Hormone receptor negative		Complete Response	Partial response	Progression	Total	P value
	High Ki-67	10	4	2	16	0.036
	Low Ki-67	5	8	9	22	
Total		15	12	11	38	

## Discussion

The evaluation of the prediction of response to chemotherapy in cancer patients has been an area of keen interest for oncologists for the past many years. It helps them in selecting the most effective chemotherapeutic regimen and thus exposing patients to minimal unwanted side effects of toxic drugs.

Many markers such as estrogen receptors, progesterone receptors and Her2/Neu receptors have been identified in breast cancer patients which provide guidance to the clinician in the selection of the most appropriate therapeutic option for them.

Ki-67 has been of particular interest in patients with breast cancer and is under investigation in many clinical trials for the evaluation of its role in the prediction of response to chemotherapy. Although the strong correlation between Ki-67 and response to chemotherapy has been established most of the studies are retrospective. There are no prospective randomized trials for this marker.

Previous studies have evaluated a sample size ranging from 70 to 1000. Mostly the patients recruited for the study had stage II and stage III breast cancer with the tumor size ranging between 2 to 5 cm and only about 4 to 16% of the patients exhibited involvement of the skin or chest wall.

Almost all the studies used Her2/Neu targeted therapy for Her2/Neu positive patients except for the Gepartrio Trial [19].

Our study conducted at the Fauji Foundation Hospital oncology department recruited 80 patients with locally advanced breast cancer. The mean age of the patients recruited in this study was 52.16 with a mean tumor size of 7.7 cm. All the patients recruited in this study had stage III disease with approximately 50% of the patients having a T 4 tumor or clinically positive lymph nodes.

The reason for 50% of our study population comprising locally advanced breast cancer is the lack of breast cancer awareness in most of the rural areas of Pakistan and therefore by the time they reach our hospital setup they have very advanced disease. Also, most of the women opt for modified radical mastectomy as there is lack of surgical expertise in lumpectomy and sentinal lymph node biopsy in many of the surgical centers in Pakistan as well as patients are lost to follow up after they have undergone surgery.

To determine the response of chemotherapy in relation to the value of Ki-67, patients in most of the studies were divided into two groups; one having a high Ki-67 value whereas the other having a low Ki-67 value. However in the Gepartrio trial patients were divided into three groups having high, intermediate and low Ki-67 values [19].

There is no standard cut-off value of Ki-67. Previous studies have either taken an arbitrary cut-off value or have taken the mean of the study population as their cut-off Ki-67 value [20]. Some studies have also taken the Saint Gallens value of Ki-67 which is used to divide luminal A and B cancers as their cut-off value and also there are a few studies that have taken out the optimal cut-off value of Ki-67 using receiver operating characteristic (ROC) curve analysis [21,22]. The range of cut-off values in these studies varies from 13 to 50% but almost every cut-off value of the studies has shown Ki-67 to be a significant independent predictor of response of chemotherapy [22,23]. Our study took 35% as the cut-off value of Ki-67 in concordance with the Gepartrio trial but due to small sample size we only divided our study population into two groups having a high and low Ki-67 value instead of three groups as was done in the Gepartrio trial. The Gepartrio trial recruited around 11600 patients of which around 30% of the study population having a Ki-67 value of more than 35% showed complete pathological response which was significant with a p value of 0.005 [19]. Another study evaluated 262 patients by analyzing data from four GEICAM clinical trials took >50% as a cut-off value of Ki-67 in neoadjuvant breast cancer settings and showed that 40% of their patients attained complete pathological response who had a Ki-67 value of more than 50% [24]. Our study population of 80 patients showed a complete pathological response of 60% in patients having a Ki-67 value of more than 35% with a significant p value of 0.00018 even though 50% of our study population had T3 and T4 tumors as compared to the Gepartrio trial having a majority of T2 tumors showing that Ki-67 is an independent predictor of response to chemotherapy in breast cancer patients.

Hormone receptor status was also evaluated in relation to Ki-67 and response in our study and the results were consistenet with the Gepartio trial showing that Ki-67 was a significant factor in determination of response in both hormone receptor positive and hormone receptor negative patients. Hormone receptor negative patients showed an increased percentage of complete pathological response especially in patients with high Ki-67 value [19].

The limitations of our study were that Her2-neu targeted therapy was not used in patients who were Her2/Neu positive and also there was no standard cut-off value of Ki-67. A larger sample size is needed to validate these results.

## Conclusions

High Ki-67 values in patients with breast cancer can correlate with attainment of complete pathological response and can be inculcated in the initial clinical assessment of the breast cancer patients to predict the effectiveness of response to chemotherapy and prevent unnecessary side effects in patients who would not respond to chemotherapy. However more prospective randomized trials are needed with a larger sample size to confirm the results. There is also a need to standardize the cut-off value of Ki-67.
